# A case report of intestinal intussusception secondary to endometriosis of the last ileal loop

**DOI:** 10.1016/j.ijscr.2025.111221

**Published:** 2025-03-27

**Authors:** Faiez Boughanmi, Mohamed Ali Chaouch, Midani Touati, Mohamed Zayati, Hiba Ben Hassine, Faouzi Noomen

**Affiliations:** Department of General Surgery, Monastir University Hospital, Tunisia

**Keywords:** Case report, Acute intestinal intussusception, Intestinal endometriosis

## Abstract

**Introduction and importance:**

Acute intestinal intussusception secondary to intestinal endometriosis is a rare entity, but it can be life-threatening. Improving diagnostic and therapeutic investigations through multidisciplinary collaboration improves its management and prognosis.

**Case presentation:**

Reporting the clinical case of a 37-year-old patient admitted for treatment of intestinal intussusception secondary to intestinal endometriosis. She underwent a resection of the invaginated segment without disinvagination and a manual end-to-end ileo-ileal anastomosis. Pathological examination concluded that there was intestinal endometriosis with simple surgical suites.

**Clinical discussion:**

Several studies have addressed the subject of intestinal intussusception secondary to intestinal endometriosis. However, the studies are mainly case reports. It is certainly rare, but potentially serious, and all studies converge on the importance of diagnosis and rapid multidisciplinary care.

**Conclusions:**

Due to its rarity and the non-specificity of its symptoms, digestive endometriosis is poorly understood and is often diagnosed late. Intussusception secondary to endometriosis is rare and generally requires surgery.

## Highlights

• Intestinal intussusception caused by endometriosis is an extremely rare condition, especially in adults. It often presents with nonspecific symptoms, making early diagnosis challenging. This case emphasizes the importance of considering intestinal endometriosis in women who present with symptoms of intestinal obstruction.
• The case highlights that surgical resection of the affected bowel segment, without attempting intraoperative disinvagination, is the standard treatment. This approach prevents the potential dissemination of endometrial tissue and serves both diagnostic and therapeutic purposes.
• Early diagnosis through imaging techniques such as abdominal ultrasound and CT scans, combined with a multidisciplinary team involving surgeons, radiologists, and pathologists, is critical to improving patient outcomes and prognosis.

## 1 Introduction

Endometriosis is an estrogen-dependent disorder that can cause substantial morbidity, including pelvic pain, multiple operations, and infertility [1]. Digestive endometriosis can cause acute complications that can be life-threatening. Although intussusception is common among children, intussusception secondary to intestinal endometriosis in an adult is rare [2]. Intestinal endometriosis complicated by intestinal intussusception can compromise the vital prognosis. However, early diagnosis is clearly improved by abdominal CT. Early multidisciplinary and surgical management improves the prognosis. Our clinical case, according to SCARE guidelines [3], shows the importance of rapid diagnosis and management of intestinal intussusception secondary to intestinal endometriosis, and a review of the literature on similar cases in order to improve the management of this rare complication.

## 2 Case presentation

**Fig. 1:**
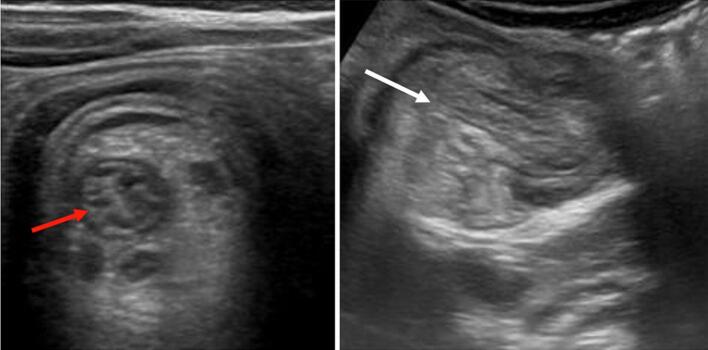
Abdominal ultrasound showing a roundel image (red arrow) and sandwich image (white arrow) showing intestinal telescoping. (For interpretation of the references to colour in this figure legend, the reader is referred to the web version of this article.)

**Fig. 2:**
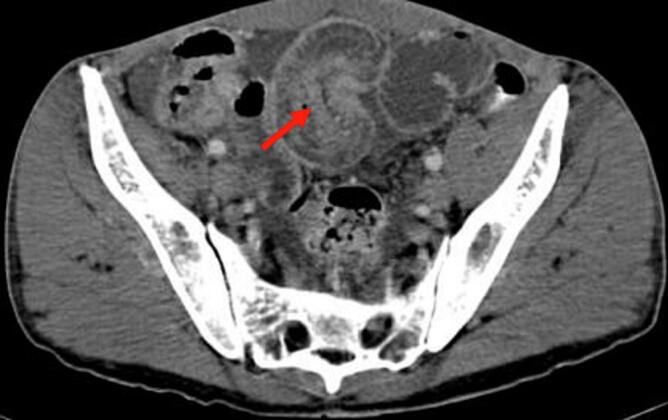
An abdominal CT scan showing the intestinal telescoping image with distention of the downstream segment (red arrow). (For interpretation of the references to colour in this figure legend, the reader is referred to the web version of this article.)

**Fig. 3:**
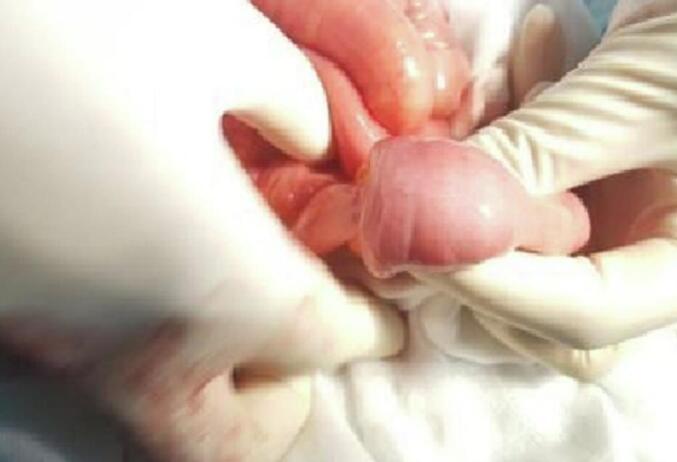
Intraoperative photos showing intestinal intussusception.

A 37-year-old patient, without a history of surgical history, was followed for three months for primary sterility. The patient did not have any documented history of pelvic inflammatory disease, nor did she report any prior symptoms suggestive of such. She has been consulting our emergency room for abdominal pain and vomiting for two days. The clinical diagnosis of acute small bowel occlusion was made. Biology did not show any inflammatory syndrome or correct biological renal function; however, it shows ionic disorders, such as hyponatremia at 130 (mEq/L). The radiological assessment found a typical image of ileo-ileal intussusception (Fig. 1, Fig. 2). Abdominal ultrasound showing a roundel image, sandwich image showing intestinal telescoping, and abdominal CT scan showing the intestinal telescoping image with distention of the downstream segment. An exploratory laparotomy revealed significant intestinal distension upstream of an ileo-ileal intussusception 10 cm from the Bauhin valve (Fig. 3). The intra-abdominal findings were non-specific and did not raise suspicion for the final histological diagnosis. There were no gross features such as abscesses, adhesions, or signs of chronic inflammation that would have suggested an underlying infectious or inflammatory etiology. We performed a resection of the affected ileal segment, including intussusception, without disinvagination and with manual end-to-end ileo-ileal anastomosis. The postoperative course was simple. Macroscopic examination of the resection specimen revealed a segment of the small intestine 15 cm in length with a proximal end more dilated than the distal end centered by intestinal telescoping, without signs of necrosis or perforation. Microscopic examination showed nests of endometriotic glands and stroma located in the muscularis propria with regional lymph node involvement. The overlying mucosa was intact. Endometrial epithelial and stromal cells were positive for the estrogen and progesterone receptors on the ileal wall and lymph nodes. Therefore, the pathological study of the surgical specimen ended with ileal endometriosis. The patient was seen in the outpoint clinic and there was no recurrence after 6 months of follow-up.

## 3 Discussion

The prevalence of endometriosis is estimated to be between 8 and 15 % of women in the period of genital activity [4]. The prevalence of digestive involvement has been estimated among women with pelvic endometriosis, between 5 and 12 % [5]. The location of digestive endometriosis is, with decreasing frequency, the recto-sigmoid (72 %), the appendix, the terminal ileum, the cecum, and the transverse colon [6]. The authors have reported that 97 % of small intestine lesions were in the last ten centimeters of the latter. The pathophysiology of intestinal endometriosis is complex. It includes several factors, such as anatomical considerations that involve invasion, fibrosis, and angiogenesis [7]. Additionally, emerging perspectives suggest a possible involvement of local neurogenesis and somatic cancer-driving mutations (KRAS), which could offer promising avenues for future therapeutic interventions [7]. Most digestive endometriosis appears to be asymptomatic. Symptoms, when present, are certain to have great variability. However, the non-specificity of the symptomatology often makes the diagnosis fortuitous; it is made on an occlusive syndrome and often during an exploratory laparotomy. As a result, treatment has changed from medical management to a multidisciplinary approach [6]. The frequency of an underlying organic lesion makes surgical treatment essential [8,9]. The risk of peritoneal dissemination of possible endometrial cells prohibits peroperative disinvagination. Resection of the affected segment without peroperative disinvagination followed by pathological examination remains a standard treatment [10]. It has the dual purpose of being diagnostic and therapeutic.

## 4 Conclusions

Due to its rarity and the non-specificity of its symptoms, digestive endometriosis is poorly understood and is often diagnosed late. Its diagnosis will be suggested in the face of functional intestinal symptoms in a context suggestive of pelvic endometriosis. In addition, digestive endometriosis can be exceptionally responsible for a complication that requires urgent surgical management. Acute intestinal intussusception in adults and endometriosis of the digestive location are uncommon pathologies, and their association remains exceptional.

## Consent

Written informed consent was obtained from the patient for the publication of this case report and accompanying images. A copy of written consent is available for review by the editor-in-chief of this journal upon request.

## Ethical approval

Not applicable.

## Funding

This research did not receive grants from the public, commercial, or non-profit sectors.

## Author contribution

All authors participated in the treatment of the patients, writing, and approving the manuscript.

## Guarantor

Mohamed Ali Chaouch

## Research registration number

1. Name of the registry: N/A.
2. Unique identifying number or registration ID: N/A.
3. Hyperlink to your specific registration (must be publicly accessible and will be checked): N/A.

## Conflict of interest statement

No conflict of interest to disclose.
